# Serum uric acid in systemic lupus erythematosus with preserved renal function: a cross-sectional and longitudinal analysis

**DOI:** 10.3389/fimmu.2026.1831052

**Published:** 2026-05-07

**Authors:** Xiaolu Huang, Hongpu Chen, Yulin Wang, Fuan Lin

**Affiliations:** Rheumatology Department of Zhangzhou Affiliated Hospital of Fujian Medical University, Zhangzhou, Fujian, China

**Keywords:** lupus nephritis, predictive biomarker, risk stratification, serum uric acid, systemic lupus erythematosus

## Abstract

**Objective:**

To evaluate the association of serum uric acid (SUA) with established lupus nephritis (LN) and to explore its prospective association with incident LN in systemic lupus erythematosus (SLE) patients presenting with preserved renal function.

**Methods:**

We enrolled 97 SLE patients with preserved renal function (creatinine clearance ≥ 90 mL/min), including 58 with baseline LN and 39 without LN (NLN). Baseline associations were evaluated using multivariable logistic regression, with a sensitivity model further adjusting for baseline glucocorticoid dose, mycophenolate mofetil use, and cyclophosphamide use. Model discrimination was compared by Area Under the Curve (AUC) and DeLong testing. The baseline NLN cohort was followed for 3 years, and incident LN was analyzed using a Cox model adjusted for baseline Systemic Lupus Erythematosus Disease Activity Index (SLEDAI).

**Results:**

Baseline SUA was higher in LN than in NLN patients (489.2 ± 80.8 vs. 339.8 ± 104.2 µmol/L, p < 0.001). Higher SUA remained associated with baseline LN in the main model (adjusted OR 4.20 per 1-SD increase, 95% CI 2.00–8.83, p < 0.001) and after additional adjustment for baseline treatment exposures (adjusted OR 6.32, 95% CI 1.21–32.96, p = 0.029). Adding SUA to the base clinical model increased discrimination within this dataset (AUC 0.863 to 0.929; DeLong test p = 0.001). During 3 years of follow-up, 11 NLN patients (28.2%) developed incident LN; in exploratory Cox analysis adjusted for baseline SLEDAI, higher baseline SUA was associated with a possible increased hazard of subsequent LN (adjusted HR 1.83, 95% CI 1.09–3.07, p = 0.022).

**Conclusion:**

In this dataset, SUA was associated with LN risk stratification in SLE patients with preserved renal function, but this signal may also reflect broader disease activity, systemic inflammation, and treatment-related factors. The renal specificity of SUA remains uncertain.

## 1 Introduction

Systemic lupus erythematosus (SLE) is a heterogeneous autoimmune disease with frequent multiorgan involvement, among which lupus nephritis (LN) remains one of the most clinically consequential manifestations (1–4). LN develops in a substantial proportion of patients during the disease course, contributes disproportionately to chronic kidney disease and kidney failure, and continues to be associated with considerable long-term morbidity and mortality despite recent therapeutic advances (2–10). Contemporary guidelines therefore emphasize the importance of early recognition, prompt risk stratification, and close longitudinal monitoring of kidney involvement in SLE (4–8).

However, the noninvasive assessment of early LN remains imperfect. Conventional surveillance tools, including serum creatinine, proteinuria, urinary sediment, complement levels, and anti-dsDNA antibodies, remain clinically indispensable but have important limitations, particularly in identifying subclinical renal inflammation or discriminating active injury from chronic damage (4–8, 11–16). Renal biopsy remains the reference standard for classification and activity assessment, yet its invasive nature and limited feasibility for repeated use have driven continued interest in blood- and urine-based biomarkers as adjunctive tools for diagnosis, activity assessment, treatment monitoring, and prognosis (7, 11–20).

This unmet need has fueled substantial work on noninvasive biomarker discovery. Recent reviews and translational studies have highlighted a growing landscape that includes conventional serologic markers, urinary chemokines, macrophage-related markers such as soluble CD163, adhesion molecules such as ALCAM and VCAM-1, and multimarker or machine-learning-based predictive models (11–25). Although many of these approaches are promising, few have yet entered routine clinical practice, and there remains a practical need for simple, inexpensive, and broadly available biomarkers that could complement standard renal assessment in everyday care (11–19).

Serum uric acid (SUA) is an attractive candidate in this context. Beyond serving as a metabolic marker, uric acid has been linked to endothelial dysfunction, oxidative stress, inflammasome activation, and tubular injury in experimental kidney research, suggesting that elevated SUA may reflect more than reduced renal clearance alone (26–28). Clinical studies have likewise associated higher SUA levels with established LN, subsequent renal damage, adverse renal prognosis, and pathologic vascular injury in LN cohorts (29–33). Nevertheless, whether SUA carries informative signal in SLE patients who still present with preserved renal function, before overt decline in conventional renal indices, remains insufficiently defined.

To address this gap, we conducted a dual-phase cohort study: cross-sectionally evaluating the association of SUA with baseline LN and assessing whether SUA improves model discrimination in this dataset, followed by a prospective longitudinal analysis on the non-LN subgroup to determine if baseline SUA is associated with incident LN. We hypothesized that elevated SUA could serve as an early indicator of renal risk prior to overt functional decline.

## 2 Materials and methods

### 2.1 Study cohort and design

This was an observational cohort study enrolling 97 consecutive SLE patients from Fujian Medical University Affiliated Zhangzhou Hospital. All patients fulfilled the SLICC and ACR classification criteria. A strict inclusion criterion was the presence of preserved renal function at baseline, defined as a creatinine clearance of at least 90 mL/min.

**Figure 1:**
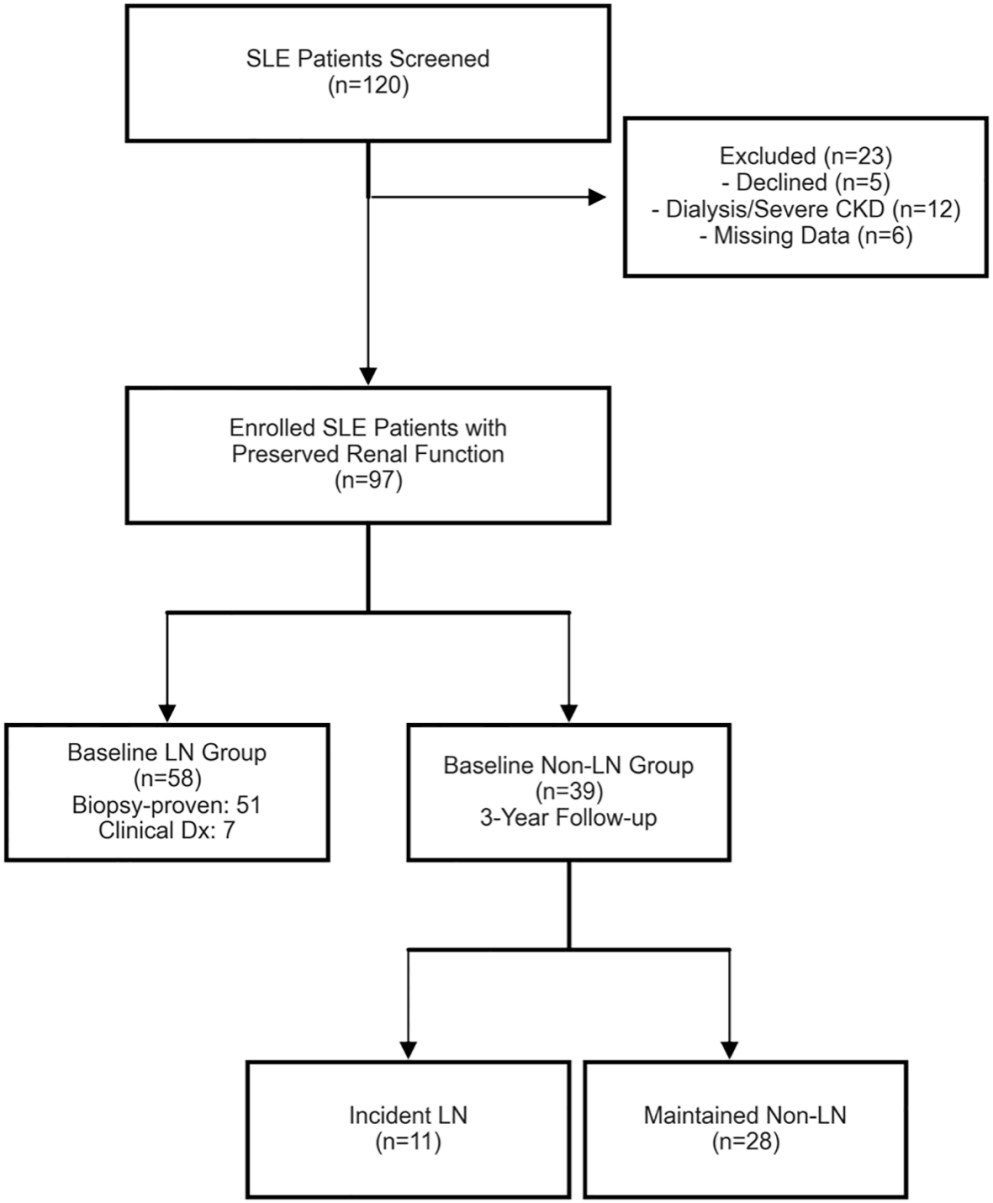
Flowchart illustrating the sequential screening, enrollment, baseline disease stratification, and longitudinal follow-up phases of the study cohort.

Initially, 120 SLE patients were screened for eligibility. Twenty-three patients were excluded due to declined participation (n = 5), presence of severe chronic kidney disease or dialysis (n = 12), or missing baseline data (n = 6), resulting in a final enrolled cohort of 97 patients (**Figure 1**).

At baseline, the cohort was stratified into an LN group (n = 58) and an NLN (non-LN) group (n = 39). Among the 58 baseline LN patients, 51 underwent renal biopsy demonstrating ISN/RPS classification classes. The remaining 7 patients were clinically diagnosed by a unified expert immunonephrology panel applying standard criteria (persistent proteinuria >>0.5 g/24 h or active urinary sediments) due to valid contraindications for biopsy. Exclusion criteria minimized potential confounders: concurrent infection, malignancy, use of drugs affecting uric acid metabolism, including urate-lowering therapy, diuretics, low-dose aspirin, calcineurin inhibitors, and other antihypertensive agents with relevant urate-modifying effects, alcohol consumption, primary gout, diabetes mellitus, severe hypertension, and overlapping autoimmune conditions. Information on some other urate-modifying medications was not systematically collected and therefore could not be further adjusted for.

For the longitudinal phase, the 39 patients in the baseline NLN group were followed prospectively over a three-year observation period to monitor for incident LN. Among the 11 incident LN cases identified, 8 were confirmed via biopsy and 3 were diagnosed clinically utilizing standard criteria by the same expert panel.

### 2.2 Ethical considerations

The study protocol complied with the Declaration of Helsinki and was approved by the local institutional ethics committee. Written informed consent was obtained from all participants.

### 2.3 Clinical and laboratory assessments

Comprehensive baseline data including demographic variables and treatment exposure (glucocorticoid dosage, hydroxychloroquine [HCQ], mycophenolate mofetil [MMF], and cyclophosphamide [CYC]) were systematically documented. Laboratory evaluations encompassed complete blood counts [white blood cell count (WBC, ×10^9^/L), hemoglobin (HGB, g/L), and platelet count (PLT, ×10^9^/L)], complement [C3 and C4 (g/L)], erythrocyte sedimentation rate (ESR, mm/h), C-reactive protein (CRP, mg/L), serum creatinine (µmol/L), 24-hour urinary protein (g/24 h), serum albumin (g/L), and serum uric acid (SUA, µmol/L). SLE disease activity was evaluated using the SLE Disease Activity Index (SLEDAI). Renal biopsies were graded for Activity and Chronicity Indices. For the supplementary sensitivity analysis, the available non-renal covariates included BMI, baseline CRP level (used here as a proxy for broader metabolic/inflammatory status), and recorded antihypertensive use; diuretic use was not systematically recorded as a separate variable and was therefore not independently incorporated into this model. Dietary purine intake, smoking, physical activity, and other detailed lifestyle variables were not systematically collected and were therefore not incorporated into the adjusted analyses. These available baseline treatment variables were further incorporated into a sensitivity analysis of the cross-sectional model.

### 2.4 Statistical analysis

Continuous variables were expressed as mean ± SD or median (range), and categorical parameters as frequencies (percentages). Group differences were tested using independent t-tests, Mann-Whitney U tests, or Chi-square/Fisher’s exact tests where appropriate.

while To evaluate the association of SUA with baseline LN while avoiding target leakage, a multivariable logistic regression model (Model A) was constructed utilizing age, sex, disease duration, SLEDAI, serum creatinine, and SUA, excluding 24-hour proteinuria which forms part of the diagnostic criteria. A sensitivity model further adjusted for baseline glucocorticoid dose, MMF use, and CYC use. Prior to modeling, Variance Inflation Factors (VIF) checked collinearity bounds (all values < 5). Odds ratios (OR) were reported per 1-standard deviation (SD) covariate increase.

Covariate selection in this prespecified main model was based primarily on clinical relevance and methodological considerations rather than purely data-driven screening: age, sex, and disease duration were included as background demographic and disease-related characteristics; SLEDAI was included to account for overall disease activity; serum creatinine was included to reflect baseline renal functional status; and SUA was the exposure variable of primary interest. Twenty-four-hour proteinuria was not included because it forms part of the diagnostic definition of LN and would therefore introduce target leakage. Treatment-adjusted and non-renal factor-adjusted analyses were performed only as supplementary sensitivity analyses to test robustness rather than replace the main model.

A supplementary pathology-oriented analysis was restricted to the 51 biopsy-confirmed baseline LN cases. Differences in SUA across histological classes were assessed using permutation testing, and correlations of SUA with Activity Index (AI) and Chronicity Index (CI) were evaluated using Spearman rank correlation.

Diagnostic discrimination was evaluated via Receiver Operating Characteristic (ROC) curve analysis. We explored changes in discrimination by comparing the Area Under the Curve (AUC) of a standard clinical model against a full model integrating SUA. To estimate the 95% Confidence Intervals (CI) for the AUCs, we employed a bootstrap resampling approach utilizing 1,000 replications. The AUCs of the base and SUA-augmented models were formally compared using the DeLong test.

For longitudinal analysis, the time to incident LN was assessed using a Cox proportional hazards regression model (Model B). To robustly accommodate the constrained event structure (n = 11) and prevent severe overfitting, the model was adjusted specifically for baseline SLEDAI rather than a full multi-variable set. Hazard Ratios (HR) and 95% Confidence Intervals (CI) were calculated. The proportional hazards assumption was assessed using Schoenfeld residuals. Additionally, incident LN-free survival was estimated using the Kaplan-Meier method, stratified by the baseline SUA median, and survival distributions were formally compared employing the unadjusted log-rank test. Computations were performed using Python (scikit-learn and lifelines) and R software. A two-tailed p < 0.05 was considered statistically significant.

This longitudinal model was not derived from data-driven variable screening; only baseline SLEDAI was retained because the limited number of incident events required active restriction of model complexity to reduce overfitting. A supplementary descriptive comparison was also performed within the baseline NLN subgroup between patients who subsequently developed incident LN and those who remained LN-free during follow-up, focusing on baseline SUA, SLEDAI, C3, C4, and CRP using the same group-comparison approach as for the other baseline variables.

## 3 Results

### 3.1 Baseline profile, pathology, and SUA

Among the 97 SLE patients with preserved renal function, the LN group (n = 58) had a mean age of 34.1 years, compared to 31.6 years in the NLN group (n = 39), p > 0.05. Notably, the proportion of patients receiving MMF and CYC was higher in the LN group, reflecting a greater expected disease burden. For the 51 biopsied baseline LN cases, distributions spanned ISN/RPS classes: II (n = 4), III (n = 14), IV (n = 23), and V (n = 10), with a mean Activity Index of 8.0 ± 3.0, Chronicity Index of 3.0 ± 2.0, and SUA of 461.5 ± 47.9 µmol/L. In an additional exploratory pathology-oriented analysis restricted to these biopsy-confirmed cases, SUA differed across histological classes (permutation p = 0.001), showed a moderate positive correlation with AI (Spearman rho = 0.433, p = 0.001), and was not significantly correlated with CI (Spearman rho = 0.009, p = 0.946).

**Figure 2:**
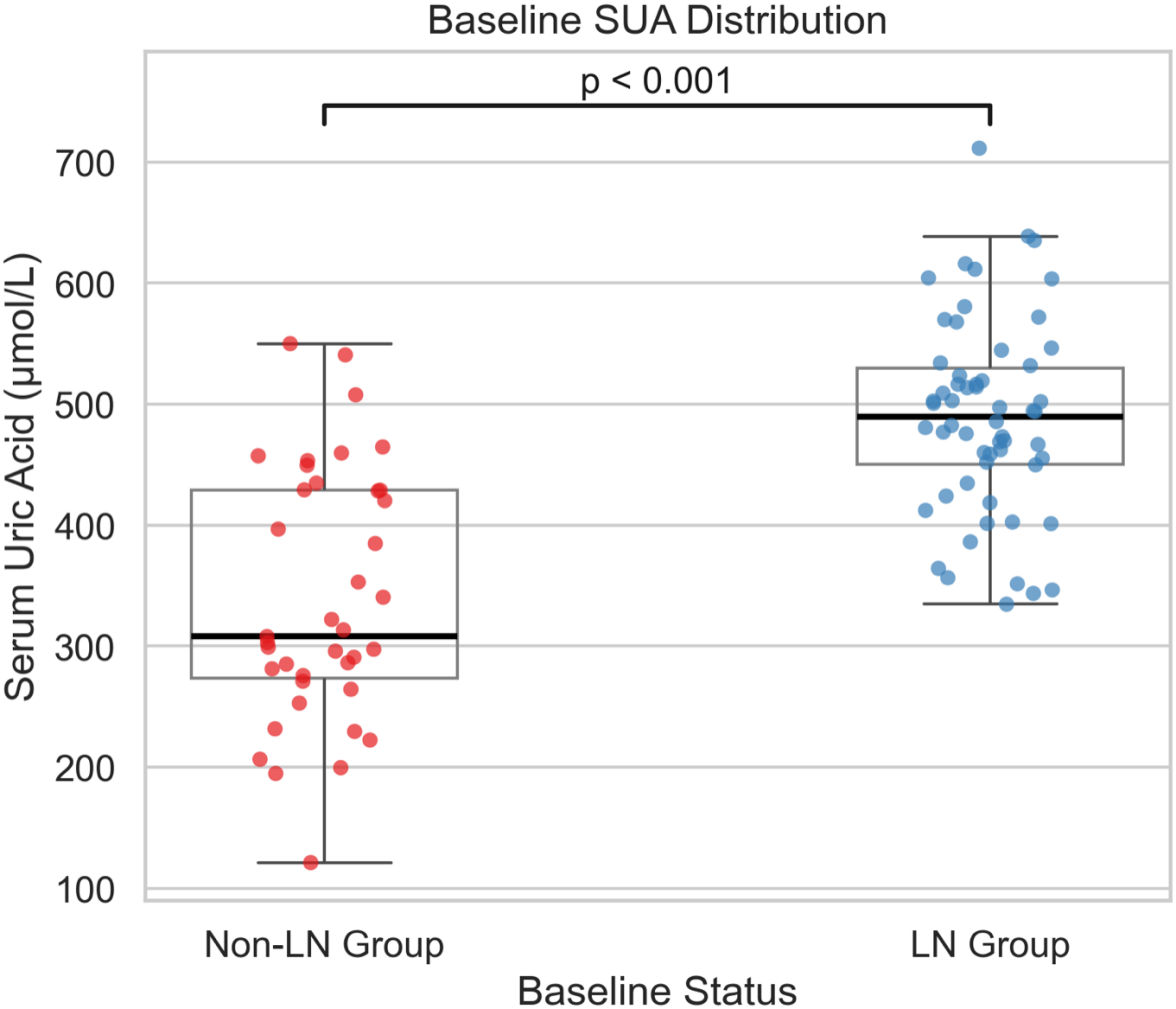
Boxplot with overlaid jittered individual data points showing baseline serum uric acid (SUA, µmol/L) levels in the lupus nephritis (LN) group and the non-lupus nephritis (NLN) group.

Baseline SUA was significantly elevated in the LN group compared with the NLN group (489.2 ± 80.8 µmol/L vs. 339.8 ± 104.2 µmol/L, p < 0.001), **Table 1**, **Figure 2**, indicating distinct uric acid elevations even in the context of globally preserved renal filtration.

**Table 1:** Baseline clinical and laboratory characteristics of the LN and NLN groups.

Characteristic	NLN group (n = 39)	LN group (n = 58)	p value
Age, years	33.4 ± 10.2	35.4 ± 12.8	0.411
Male sex, n (%)	5 (12.8%)	9 (15.5%)	0.939
Disease duration, months	69.5 ± 58.7	84.3 ± 68.9	0.277
WBC, ×10^9^/L	4.4 ± 2.8	5.5 ± 2.5	**0.049**
HGB, g/L	131.1 ± 13.7	111.1 ± 21.2	**<0.001**
PLT, ×10^9^/L	218.1 ± 45.0	187.4 ± 77.4	**0.028**
C3, g/L	0.6 ± 0.3	0.5 ± 0.3	0.078
C4, g/L	0.1 ± 0.1	0.1 ± 0.1	**0.012**
ESR, mm/h	45.2 ± 27.9	43.3 ± 20.8	0.700
CRP, mg/L	13.6 ± 12.8	4.7 ± 3.9	**<0.001**
Creatinine, µmol/L	54.8 ± 15.7	74.6 ± 18.2	**<0.001**
Albumin, g/L	40.3 ± 5.0	29.0 ± 9.1	**<0.001**
Proteinuria, 24 h, g/24 h	0.2 ± 0.1	3.1 ± 3.4	**<0.001**
SLEDAI	9.5 ± 3.2	12.8 ± 3.0	**<0.001**
SUA, µmol/L	339.8 ± 104.2	489.2 ± 80.8	**<0.001**
Glucocorticoid dose, mg/day	16.5 ± 8.2	27.3 ± 12.4	**<0.001**
HCQ, n (%)	32 (82.1%)	47 (81.0%)	1.000
MMF, n (%)	9 (23.1%)	30 (51.7%)	**0.009**
CYC, n (%)	0 (0.0%)	28 (48.3%)	**<0.001**

Data are presented as mean ± SD for continuous variables and n (%) for categorical variables. LN, lupus nephritis; NLN, non-lupus nephritis; SUA, serum uric acid; SLEDAI, Systemic Lupus Erythematosus Disease Activity Index; HCQ, hydroxychloroquine; MMF, mycophenolate mofetil; CYC, cyclophosphamide; WBC, white blood cell count; HGB, hemoglobin; PLT, platelet count; ESR, erythrocyte sedimentation rate; CRP, C-reactive protein. Bold values indicate statistical significance, i.e., P < 0.05.

### 3.2 Multivariable association and model discrimination

**Figure 3:**
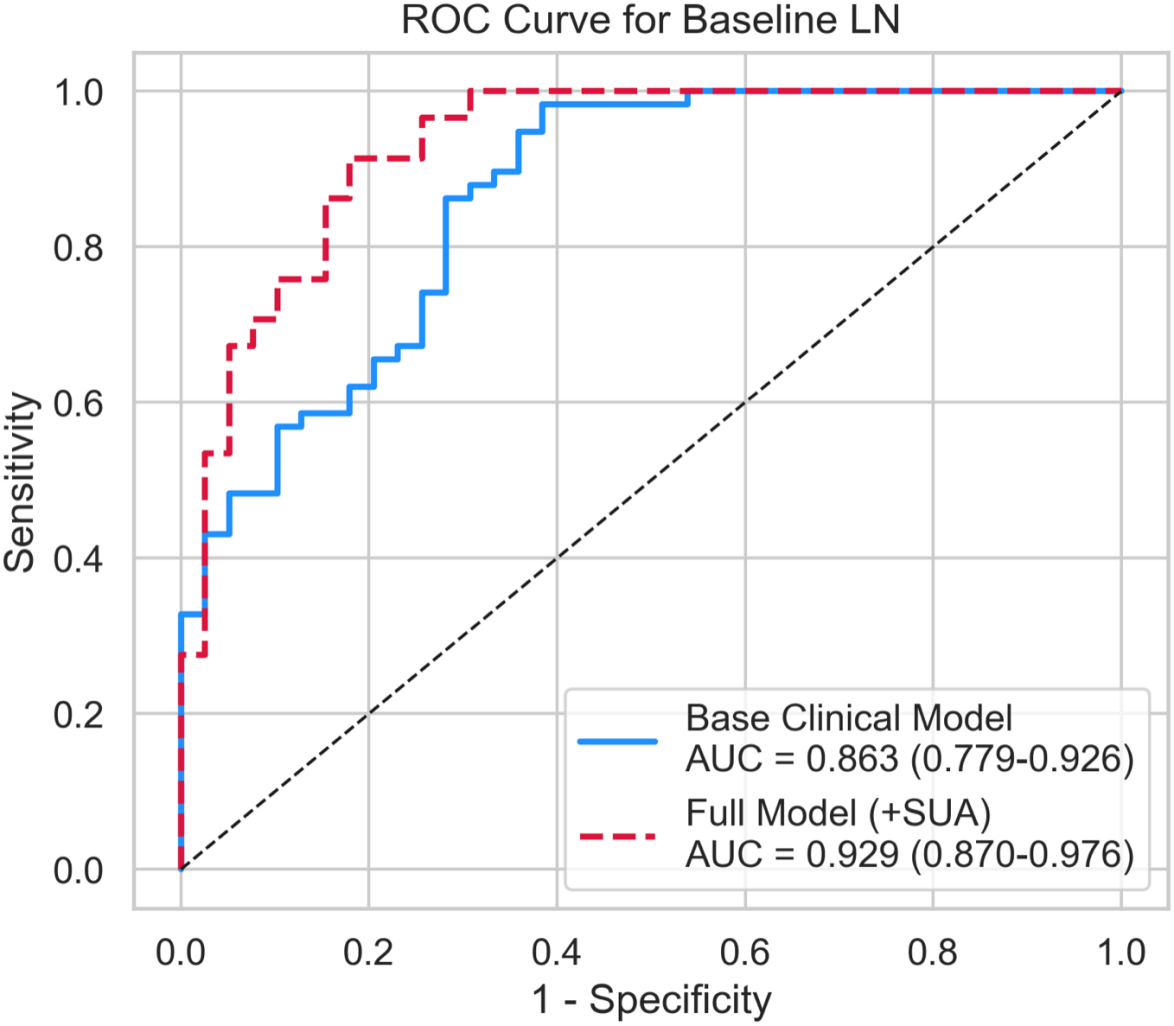
Forest plots illustrating the adjusted effect sizes and 95% confidence intervals (95% CI) for both the multivariable logistic baseline lupus nephritis (LN) model (Model A; Panel A, odds ratios) and the prospective incident LN Cox model (Model B; Panel B, hazard ratios). All effect sizes for continuous variables are calculated per 1-SD increment.

To assess the association of SUA with baseline LN after adjustment for clinical covariance without introducing target leakage, a multivariable logistic regression (Model A) was performed after excluding elements of the diagnostic criterion (e.g., 24-hour proteinuria). Even when accounting for these classical markers, elevated SUA remained associated with LN status (Adjusted OR = 4.20 per 1-SD increase, 95% CI 2.00–8.83, p < 0.001, **Table 2**; **Figure 3**). In a supplementary sensitivity analysis further adjusting for BMI, baseline CRP level, and recorded antihypertensive use, the SUA-LN association remained directionally consistent and materially stable (Adjusted OR = 8.93 per 1-SD increase, 95% CI 2.38–33.51, p = 0.001). In a sensitivity analysis further adjusting for baseline glucocorticoid dose, MMF use, and CYC use, the association remained directionally consistent (Adjusted OR = 6.32 per 1-SD increase, 95% CI 1.21–32.96, p = 0.029).

**Table 2:** Multivariable logistic regression for baseline LN status.

Covariate	Adjusted OR (95% CI)*	p value
Age	1.20 (0.63 - 2.28)	0.588
Male sex	1.11 (0.62 - 1.98)	0.730
Disease Duration, months	0.93 (0.54 - 1.61)	0.794
SLEDAI	2.41 (1.22 - 4.73)	**0.011**
Creatinine	2.94 (1.46 - 5.94)	**0.003**
SUA	4.20 (2.00 - 8.83)	**<0.001**

Odds ratios were calculated per 1-SD increase for continuous variables. LN, lupus nephritis; SLEDAI, Systemic Lupus Erythematosus Disease Activity Index; SUA, serum uric acid; OR, odds ratio; CI, confidence interval; SD, standard deviation. Bold values indicate statistical significance, i.e., P < 0.05.

**Figure 4:**
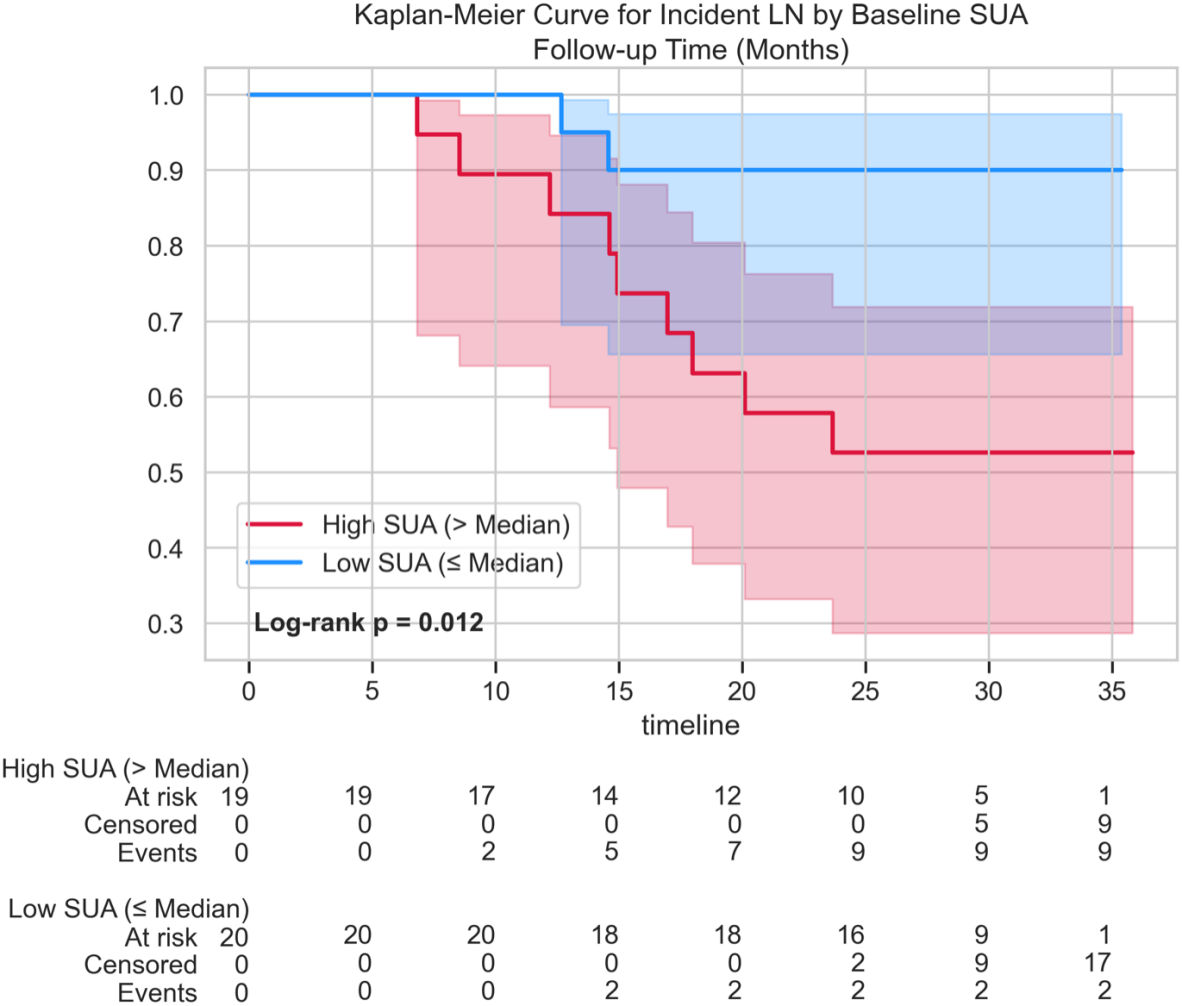
Receiver operating characteristic (ROC) curves with the area under the curve (AUC) and 95% bootstrap confidence intervals, comparing the base model and the serum uric acid (SUA)-augmented model in this dataset.

In evaluating diagnostic performance, the base clinical model yielded an AUC of 0.863. The full model incorporating SUA yielded a noticeably higher AUC of 0.929 (**Figure 4**). While promising, this improvement relies on a single dataset and must be interpreted cautiously absent external validation. The DeLong test indicated that the AUC difference was statistically significant within this dataset (p = 0.001).

### 3.3 Longitudinal validation: predicting incident LN

Over the three-year prospective span, 11 of the 39 baseline NLN patients (28.2%) progressed to incident LN. This longitudinal observation permitted evaluating baseline SUA as an exploratory risk signal. In a descriptive comparison within the baseline NLN subgroup, patients who subsequently developed incident LN had higher baseline SUA levels than those who remained LN-free during follow-up, whereas no clear between-group differences were observed for SLEDAI, C3, C4, or CRP (**Supplementary Figure 1**).

**Figure 5:**
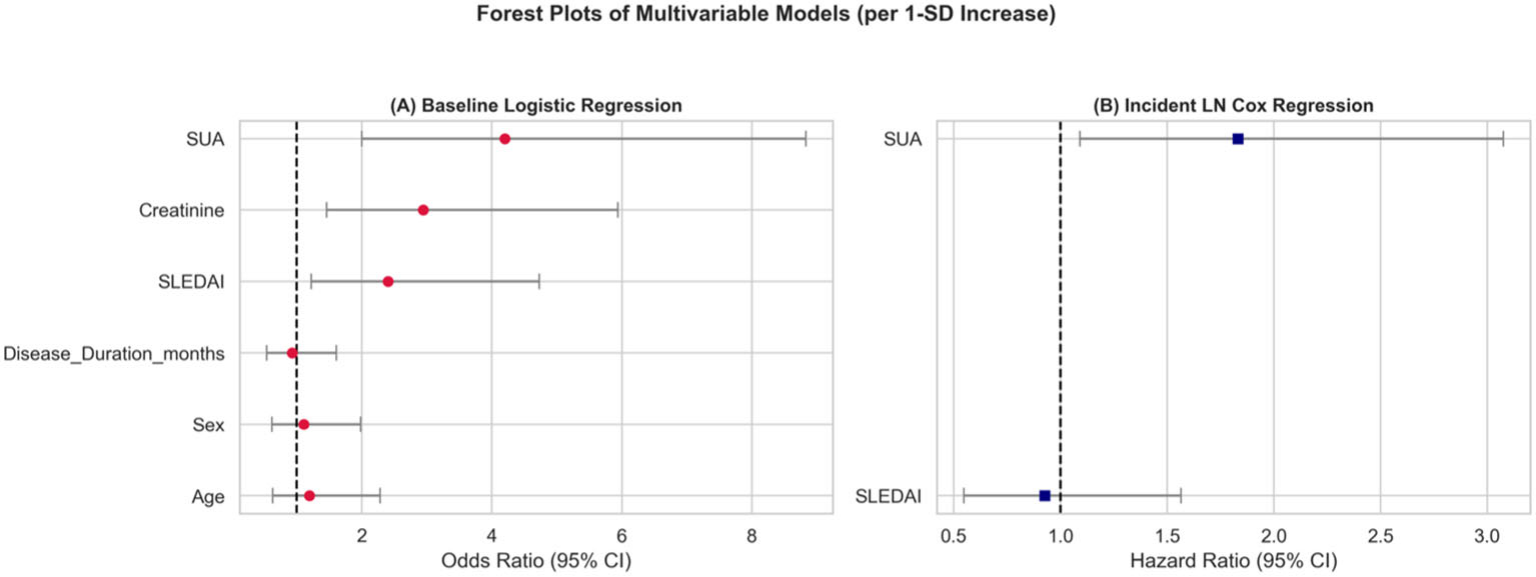
Kaplan-Meier curve stratifying incident lupus nephritis (LN)-free survival by the baseline cohort median serum uric acid (SUA) level (high SUA: > median; low SUA: ≤ median). Includes number-at-risk table and unadjusted log-rank comparison.

Analyzed via a Cox regression model adjusted for baseline disease activity (Model B) to minimize overfitting with the constrained event count, baseline SUA suggested a possible association with subsequent LN development. Each 1-SD increase in baseline SUA suggested a possible increased hazard of developing overt LN (Adjusted HR = 1.83, 95% CI 1.09–3.07, p = 0.022, **Table 3**; **Figure 5**). This finding is consistent with SUA serving as an exploratory early risk signal. Schoenfeld residual testing did not indicate a clear violation of the proportional hazards assumption.

**Table 3:** Cox regression for incident LN (adjusted for baseline disease activity).

Covariate	Adjusted HR	95% CI	p value
SLEDAI	0.93	0.55 - 1.56	0.774
SUA	1.83	1.09 - 3.07	**0.022**

Hazard ratios were calculated per 1-SD increase for continuous variables. LN, lupus nephritis; SLEDAI, Systemic Lupus Erythematosus Disease Activity Index; SUA, serum uric acid; HR, hazard ratio; CI, confidence interval; SD, standard deviation. Bold values indicate statistical significance, i.e., P < 0.05.

## 4 Discussion

In this observational cohort study of SLE patients meeting the preserved renal function threshold by study definition (CrCl ≥ 90 mL/min), SUA was associated with baseline LN and showed an exploratory prospective association with the subsequent development of incident LN over a 3-year follow-up. Additionally, ROC analysis suggested a dataset-specific improvement in model discrimination when SUA was added to traditional clinical variables. A sensitivity analysis further adjusting for baseline glucocorticoid dose, MMF use, and CYC use showed a directionally consistent association between SUA and baseline LN.

These findings align with an accumulating body of literature suggesting that SUA is clinically relevant in LN. Prior cohort studies have linked higher SUA to active LN, new-onset renal damage, poorer kidney outcomes, long-term renal prognosis, sex-specific progression risk, and renal arteriolopathy in biopsy-characterized LN populations (29–33). Our study extends that signal into a narrower and clinically important setting by focusing on patients who still met a preserved renal function threshold at study entry. In that context, the observed association between SUA and both baseline LN and subsequent incident LN suggests that SUA may retain informative value even among patients with preserved renal function at baseline. In the biopsy-confirmed subgroup, the supplementary pathology-oriented analysis further suggested that SUA varied across histological classes and was more closely aligned with pathological activity than with chronic damage, because SUA correlated with AI but was not significantly correlated with CI; however, these subgroup findings remain exploratory and should be interpreted cautiously.

Importantly, all participants in this study met the inclusion threshold of creatinine clearance ≥ 90 mL/min and therefore fell within a cohort with preserved overall renal function by study definition. However, serum creatinine was still significantly higher in the LN group than in the NLN group, indicating that SUA may partly reflect early or mild renal functional variation despite the preserved creatinine clearance threshold.

In our dataset, patients with LN also had higher SLEDAI scores, together with complement-related differences, especially lower C4 levels, with a lower C3 trend, and greater exposure to glucocorticoids, MMF, and CYC, and less favorable laboratory parameters including lower albumin and hemoglobin. Accordingly, elevated SUA in this cohort may reflect not only renal risk-related processes but also broader disease activity, systemic inflammation, and treatment-related effects. Within the baseline NLN subgroup, patients who later developed incident LN showed higher SUA levels at study entry, whereas the available disease-activity and immune-related indicators did not show clear between-group differences in this small exploratory comparison. This pattern suggests that baseline SUA may still capture a risk-related signal in the absence of a clearly separable immune-inflammatory profile in the available subgroup data. Within the constraints of the present dataset, SUA is therefore more appropriately interpreted as an integrated clinical signal associated with LN risk stratification rather than a renal-specific biomarker.

The reversed CRP pattern also warrants cautious interpretation. CRP is not always a stable or specific indicator of lupus nephritis status in SLE, and CRP levels may be influenced by non-renal inflammatory processes, individual biological heterogeneity, and short-term inflammatory variation. In this cohort, the reversed between-group CRP difference suggests inflammatory heterogeneity, and no single inflammatory marker should therefore be used in isolation to represent LN risk.

At the same time, our results should be interpreted within the broader LN biomarker landscape. Current evidence indicates that traditional markers alone incompletely capture renal inflammatory activity, while numerous noninvasive candidates, including soluble CD163, ALCAM, VCAM-1, MCP-1, PF4, and multimarker panels, have shown promise for diagnosis, histologic activity assessment, treatment response monitoring, and outcome prediction (11–25). However, most of these biomarkers remain limited by assay availability, cost, standardization challenges, or incomplete external validation (11–19). In this specific context, SUA is not proposed here as a replacement for established or emerging biomarkers, but rather as a routine laboratory test that can be evaluated alongside standard clinical assessments. In this setting, SUA is more appropriately interpreted as an integrated clinical signal rather than a renal-specific biomarker.

The biological plausibility of this association is also supported by prior mechanistic work. Uric acid has been implicated in endothelial dysfunction, vascular inflammation, oxidative stress, tubular phenotypic transition, and NLRP3-related inflammatory signaling in kidney injury models (26–28). These pathways provide a coherent framework through which elevated SUA might reflect a renal microenvironment characterized by early inflammatory and vascular stress rather than simply reduced filtration. In addition, the current treatment and pathogenesis literature increasingly emphasizes the close interconnection between innate immune activation, intrarenal inflammation, tissue remodeling, and long-term outcome in LN (2–8, 14, 18, 21). Our findings are compatible with the possibility that SUA may act as a downstream clinical correlate of those broader processes. Accordingly, SUA may represent an integrated clinical signal influenced by renal risk, systemic inflammation, disease severity, treatment exposure, baseline CRP level, and unmeasured lifestyle factors.

The potential therapeutic implication of hyperuricemia also warrants cautious interpretation. Because the present study was observational, it can support only an association between SUA and LN risk stratification rather than a treatment effect. Accordingly, our data do not establish that lowering SUA would improve renal survival or prevent subsequent renal involvement. Although prior mechanistic work suggests that uric acid may participate in inflammatory, endothelial, and renal injury pathways, whether urate-lowering therapy can modify renal outcomes in SLE/LN remains a question for future prospective interventional studies.

A key practical advantage of SUA lies in its routine clinical availability. Unlike many emerging urine or multi-omics biomarkers, SUA is inexpensive, standardized, and routinely measured in almost all clinical laboratories. If validated in larger prospective cohorts, SUA could be incorporated into renal surveillance frameworks as a low-cost adjunct marker for identifying apparently non-nephritic SLE patients who may warrant closer follow-up or more careful re-evaluation (4–8, 11–19). This would be particularly relevant in settings where access to repeat biopsy or advanced biomarker testing is limited.

These findings must be interpreted alongside important methodological limitations. First, this remains a single-center cohort with a restricted sample frame. Crucially, the occurrence of only 11 incident events severely limits statistical power and increases the risk of overfitting. Although we restricted the number of model correlates to mitigate this, the hazard ratio observed here (HR 1.83, p = 0.022) still relies on a constrained sample size and must be treated as strictly hypothesis-generating. Even with this restricted covariate adjustment strategy, the HR estimate may remain unstable because only 11 incident LN events were observed. Second, the AUC increase from 0.863 to 0.929 after adding SUA must similarly be viewed with caution. This finding merely reflects improved discrimination specifically in this dataset, and the lack of external validation means the performance is likely overly optimistic. Although the DeLong test suggested a statistically significant difference within this dataset (p = 0.001), this estimate may still be affected by optimism bias in a small single-center cohort. Moreover, the present study did not include a full calibration assessment, so the overall predictive performance of the model cannot be judged from AUC alone. Therefore, the observed AUC gain should be interpreted only as a dataset-specific improvement in discrimination rather than a stable, generalizable model advantage. Since AUC improvements represent only one facet of discriminative capacity, a comprehensive validation of specific clinical utility will require more rigorous internal validation, external validation, formal assessment of both discrimination and calibration, and decision-analytic evaluations such as Net Reclassification Improvement (NRI) or Decision Curve Analysis (DCA) (17, 18). Third, treatment changes during follow-up may have introduced time-varying confounding, but the available sample size and event count were insufficient to support reliable time-dependent modeling or more complex dynamic adjustment. In addition, patients with LN received more intensive glucocorticoid and immunosuppressive treatment, which may have introduced confounding by indication; although the baseline sensitivity analysis further adjusted for glucocorticoid dose, MMF use, and CYC use, residual treatment-related confounding cannot be fully excluded. Given the limited number of incident events, this issue could not be reliably modeled in the longitudinal analysis. Therefore, the longitudinal findings should still be interpreted as exploratory and hypothesis-generating. Finally, direct head-to-head comparisons between SUA and emerging urine biomarkers were not available (11–25). In addition, dietary purine intake, smoking, physical activity, and other detailed lifestyle or behavioral metabolic factors were not systematically collected in this cohort, and diuretic use was not systematically recorded as a separate variable. Therefore, we could not fully distinguish whether elevated SUA reflected early renal risk or broader metabolic, pharmacologic, and environmental influences, and its renal specificity remains uncertain. Elevated SUA may also reflect broader disease activity and systemic inflammatory burden rather than renal involvement alone, and the present study could not fully separate a renal risk-related signal from a global disease severity signal.

## 5 Conclusion

In conclusion, within this single-center cohort of SLE patients with preserved renal function, SUA was associated with LN risk stratification in this dataset. However, this signal may reflect not only renal risk but also broader disease activity, systemic inflammation, treatment exposure, and other metabolic or lifestyle-related influences. Therefore, the renal specificity of SUA remains uncertain and requires further validation in cohorts with more comprehensive clinical, metabolic, and lifestyle profiling. Whether lowering SUA can improve renal outcomes remains uncertain and should be tested in future prospective interventional studies.

## Data Availability

The raw data supporting the conclusions of this article will be made available by the authors, without undue reservation.
