# Supplementary material for: Serum uric acid in systemic lupus erythematosus with preserved renal function: a cross-sectional and longitudinal analysis
Source: Front Immunol. 2026 May 7;17:1831052. doi: 10.3389/fimmu.2026.1831052 (PMC13189868; doi:10.3389/fimmu.2026.1831052)
Supplement: Supplementary file 1 [file Table1.docx]

Supplementary Material

## Supplementary Figures


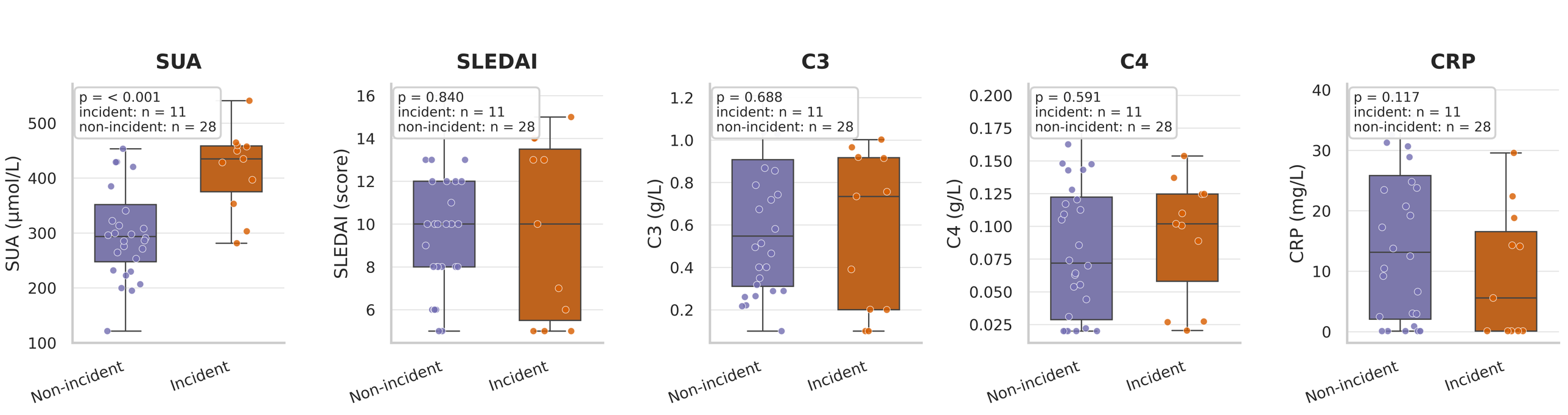


Supplementary Figure 1. Baseline comparison within the non-lupus nephritis subgroup between patients with and without subsequent incident lupus nephritis. Baseline non-lupus nephritis (NLN) subgroup only. The figure displays baseline serum uric acid (SUA), Systemic Lupus Erythematosus Disease Activity Index (SLEDAI), complement C3, complement C4, and C-reactive protein (CRP) for patients who later developed incident lupus nephritis (LN) and those who remained LN-free during follow-up. This comparison is descriptive and exploratory.
